# Exploring biomarkers of MAPK pathway co-expression in lung adenocarcinoma and their functions based on machine learning algorithms and single-cell analysis

**DOI:** 10.1016/j.gendis.2024.101222

**Published:** 2024-01-26

**Authors:** Mingkai Lin, Ruoyi Zheng, Peixian Liang, Jiayang Huang, Xintong Ke, Wenjing Zhang, Pei Shang

**Affiliations:** a Breast Center, Department of General Surgery, Nanfang Hospital, Southern Medical University, Guangzhou, Guangdong 510515, China; b Department of Ultrasound, The Second Affiliated Hospital of Guangzhou Medical University, Guangzhou, Guangdong 510515, China; c College of Teacher's Education, Guangdong University of Education, Guangzhou, Guangdong 510515, China; d Department of Respiratory Medicine, Puyang People's Hospital, Henan 457000, China

Nowadays, although the treatment and diagnostic approaches have been improved, lung adenocarcinoma (LUAD) is still the leading cause of cancer-related death in the world with overall survival of less than five years^1^. Diagnosis of LUAD in the early stage is still a challenge, resulting from that early symptoms are not obvious^2^, which leads to the fact that most patients eventually die from cancer progression and chemotherapy resistance. LUAD is reported to be associated with the MAPK pathway, however, the mechanism at the cellular and genetic levels has not been clearly elucidated. Therefore, functional exploration of LUAD- and MAPK pathway-related genes is essential for understanding the pathogenesis of LUAD and exploring therapeutic measures. Figure S1 illustrates the flowchart of this study.

First, to identify potential biomarkers for this disease, the GEO (http://www.ncbi.nlm.nih.gov/geo/) database was systematically searched and consequently, the microarray data of GSE116959 were identified and downloaded, involving 57 LUAD samples and 11 normal controls. Next, software R (version 4.2.2, https://www.r-project.org/) and the “limma” package were used to perform the significance analysis of differentially expressed genes (DEGs) between LUAD samples and normal samples, and then 731 genes with adjusted *P*-value < 0.05 and |Log2 (fold change)| > 1 were considered as DEGs. These DEGs were presented in a volcano plot (Fig. S2A) and a heat map of the top 50 DEGs was plotted (Fig. S2B).

A gene co-expression network was constructed using the “WGCNA” package in R software and 1747 MAPK pathway co-expressed genes from GSE116959 were obtained. In WGCNA^3^, the scale-free fit index was set to 0.9 to respectively obtain a minimum soft threshold of 6 for constructing the scale-free networks (Fig. S2C). The minimum number of genes in the modules is set to 50, and 26 modules were obtained (Fig. S2D). The MEbrown module in LUAD samples showed a strong correlation with both LUAD and MAPK pathway (Fig. S2E). Ultimately, we took intersections of MEbrown module genes and DEGs to generate 413 targets associated with both LUAD and MAPK pathway (Fig. S2F). These 413 genes were imported into the String database (https://cn.string-db.org) to obtain the protein-protein interaction network composed of 211 nodes and 5538 edges and this network was visualized through Cytoscape 3.7.2 software^4^ (Fig. S3A). Then 26 genes with “degree ≥ 10” in the protein-protein interaction network were selected for survival analysis showing that seven genes (GNAI1, ITGB4, KRT8, PDGFB, PECAM1, PIK3R1, and YWHAZ) with high or low expression had a statistically significant effect on the survival time (Figs. S3B–H).

LASSO analysis yielded seven candidate biomarkers with the model in λ analysis accurately predicting LUAD when *λ* = 7 (Fig. 1A). Meanwhile, five genes with “MeanDecreaseGini” > 2 were selected from random forest analysis (Fig. 1B). Finally, the results of both algorithms were combined to conclude that KRT8, PDGFB, PECAM1, PIK3R1, and YWHAZ were potential biomarkers for LUAD (Fig. 1C), which might contribute to future research in this domain. Data of receiver operating characteristic analyses obtained from the original dataset GSE116959 (area under the receiver operating characteristic curve was 0.936, 0.938, 0.989, 0.941, and 0.869, respectively) and validated in the TCGA database (area under the receiver operating characteristic curve was 0.884, 0.871, 0.995, 0.897, and 0.920, respectively) were demonstrated in Figure S4A and B.

**Figure 1:**
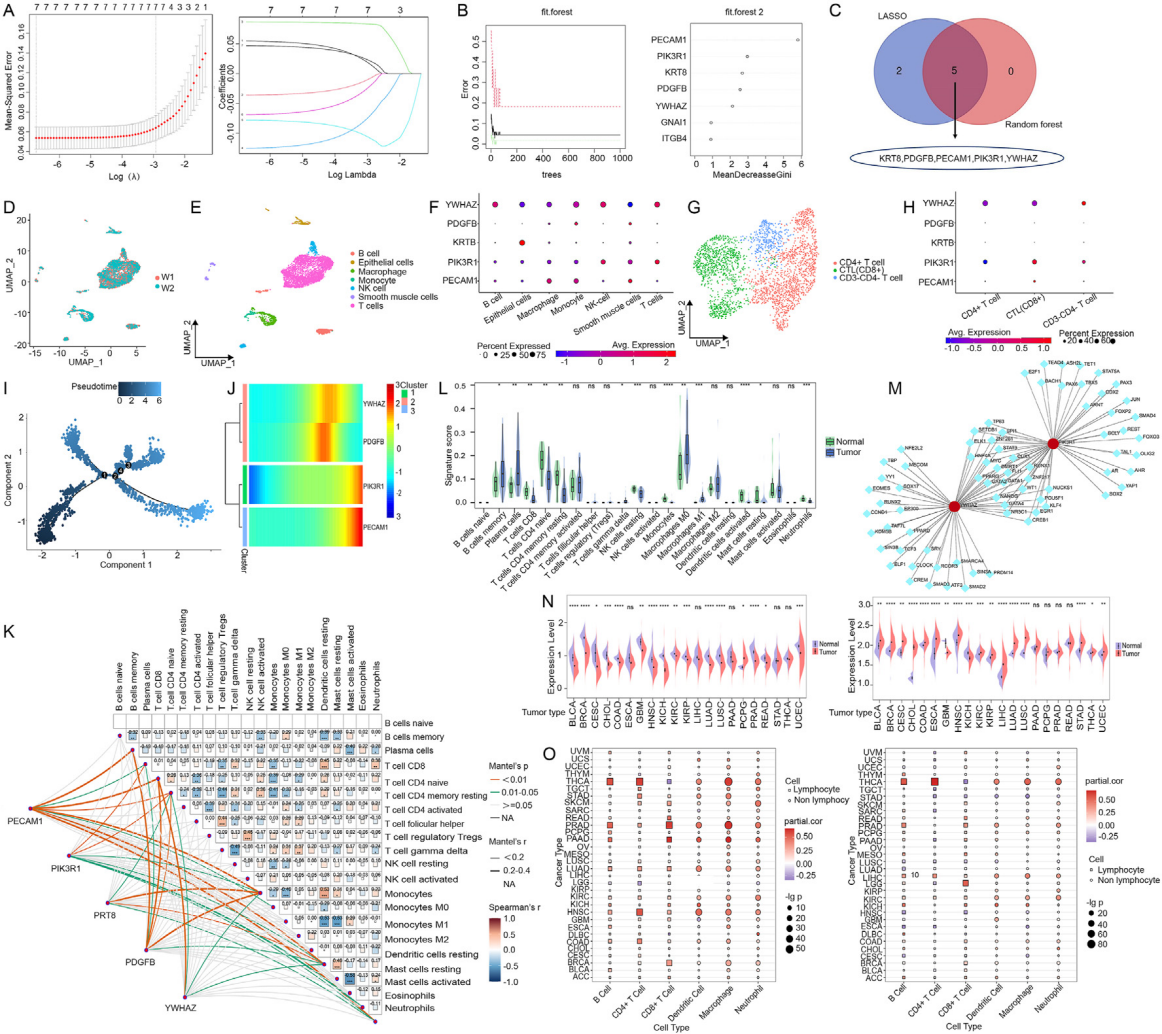
Identification of pot ential biomarkers for lung adenocarcinoma and functional analysis of immune-related genes. **(A)** LASSO regression model. **(B)** The random forest model (left) and the seven genes in terms of importance (random forest model) (right). **(C)** The intersection of the results of the two machine learning algorithms. **(D)** Visualization of cells after removal of batch effects. **(E)** Cell subcluster identification using singleR. **(F)** Expression of potential biomarkers in different cell types. **(G)** Subpopulations of T cells identified by marker genes. **(H)** Expression of potential biomarkers in different types of T cells. **(I)** The Monocle 2 trajectory plot showing the dynamics of T cell subclusters. **(J)** Four potential biomarkers varied significantly along the pseudotime (qval <0.1). **(K)** Heatmap of correlation in 21 types of immune cells (naive B cells were eliminated due to the absence of infiltration). The red represented a positive correlation, while the blue represented a negative correlation. The darker color indicated a stronger association. **(L)** Box plot for immune cell infiltration analysis. The blue represented the LUAD group and the green represented the control group. ns, no significant; LUAD, lung adenocarcinoma. **(M)** TF-mRNA network. The red circles represented mRNAs and the blue squares represented transcription factors. **(N)** Pan-cancer analysis of PIK3R1 and YWHAZ expression in tumors and normal tissues (above: PIK3R1; below: YWHAZ). **(O)** Pan-cancer analysis of PIK3R1 and YWHAZ expression and immune cell infiltration (left: PIK3R1; right: YWHAZ). ns, no significant; ∗*P* < 0.05; ∗∗*P* < 0.01; ∗∗∗*P* < 0.001; ∗∗∗∗*P* < 0.0001.

The single-cell RNA sequencing data were obtained from 6109 cells from the two LUAD samples (W1 and W2) of GSE146100. Next, we normalized the data and selected the top 2000 highly variable genes (Fig. S5A), used the PCA method and the Harmony package for dimensionality reduction and removal of batch effects (Fig. 1D) and divided the cells into 15 clusters (Fig. S5B). After annotating the cells using the SingleR package, cells were divided into seven subclusters, namely B cells, epithelial cells, macrophages, monocytes, natural killer cells, smooth muscle cells, and T cells. Then, we analyzed the expression of potential biomarkers KRT8, PDGFB, PECAM1, PIK3R1, and YWHAZ in them (Fig. 1E). The results showed that PECAM1 was more highly expressed in monocytes, macrophages, and smooth muscle cells, PIK3R1 in T cells, KRT8 in epithelial cells, and YWHAZ in lymphocytes (Fig. 1F).

T cell subcluster annotation was performed and T cells selected from the W1 samples were classified into three types via recognized marker genes^5^ (Fig. S5D), namely cytotoxic T cells, CD4 T cells, and CD3-CD4- T cells (Fig. S5C, 1G). Figure 1H demonstrates the differential expression of potential biomarkers in the three types of T cells, with PIK3R1 being more highly expressed in cytotoxic T cells and YWHAZ being more highly expressed in CD3-CD4- T cells on average. The dimensionality of T cells was reduced based on highly variable genes (Fig. S5E) and the trajectories of T cell distribution with pseudotime were visualized (Fig. 1I). The trajectory analysis yielded four crucial time nodes, by which the T cells were divided into nine cell states (Fig. S5F). We found that the expression of PIK3R1 and YWHAZ was significantly higher than other marker genes in the three types of T cells, and with the change of cell states, these two genes also showed significant changes, especially PIK3R1 (Fig. S5G). In addition, the expression of PIK3R1 and PECAM1 gradually increased with T cell trajectory, and the expression of YWHAZ and PDGFB was elevated in the middle to late stages of T cell trajectory (Fig. 1J).

In our study, we estimated the proportion of 22 immune cells in 57 LUAD samples and 11 control samples with the CIBERSORT algorithm (Fig. S6A) and analyzed the correlation of immune cell infiltration in the tissues (Fig. 1K). Then, immune cell infiltration in LUAD tissues and control samples were compared in Figure 1L. The results showed that there were differences in the proportions of many types of immune cells. Due to the high expression of PIK3R1 and YWHAZ in lymphocytes, we focused on the immunological functions of these two genes. Correlation analysis of PIK3R1 and YWHAZ expression showed a significant correlation (R = −0.2, *P* = 3.9E-06) (Fig. S6B). The NetworkAnalyst platform (https://www.networkanalyst.ca) was used to construct the TF-mRNA network, which revealed that there are many common transcription factors between PIK3R1 and YWHAZ, explaining why these two genes are expressed in association (Fig. 1M).

We performed a pan-cancer analysis of PIK3R1 and YWHAZ which were statistically analyzed and visualized with R software based on data from the UCSC database (Fig. 1N), showing that PIK3R1 was significantly down-regulated in 15 types of tumors, such as bladder urothelial carcinoma and breast invasive carcinoma, and significantly up-regulated in pheochromocytoma and paraganglioma. YWHAZ was significantly up-regulated in 15 types of tumors, such as breast invasive carcinoma and stomach adenocarcinoma, and significantly down-regulated in two types of tumors. Based on the TIMER platform, PIK3R1 has the strongest association with thyroid carcinoma, and notably, YWHAZ also correlated strongly with thyroid carcinoma in immune cell infiltration (Fig. 1O).

In the current study, five potential biomarkers for LUAD were identified and validated, and that these genes were strongly associated with the survival time of the patients. Focusing on the expression characterization and immunological functions of PIK3R1 and YWHAZ, we found that PIK3R1 and YWHAZ play a non-negligible role in the maturation of T cells, a role that may also be present in a variety of cancers.

## Ethics declaration

This study did not include any research on animals. All methods were performed in accordance with relevant guidelines and regulations.

## Author contributions

Mingkai Lin and Shang Pei contributed to the conception and planning of the project. Mingkai Lin and Peixian Liang performed data analysis and data visualization. Mingkai Lin, Ruoyi Zheng, and Jiayang Huang wrote the manuscript. Xintong Ke and Wenjing Zhang were responsible for organizing the data and charts. All authors reviewed the manuscript. Shang Pei and Wenjing Zhang revised and integrated the manuscript.

## Conflict of interests

The authors declared that they had no conflict of interests.

## Funding

This research was supported by the Guangzhou basic and applied basic research project (China) (No. 2023A04J2337).

## Data availability

Data supporting the reported results may be found in the article and supplementary information.

## Figures and Tables

**Figure S1:**
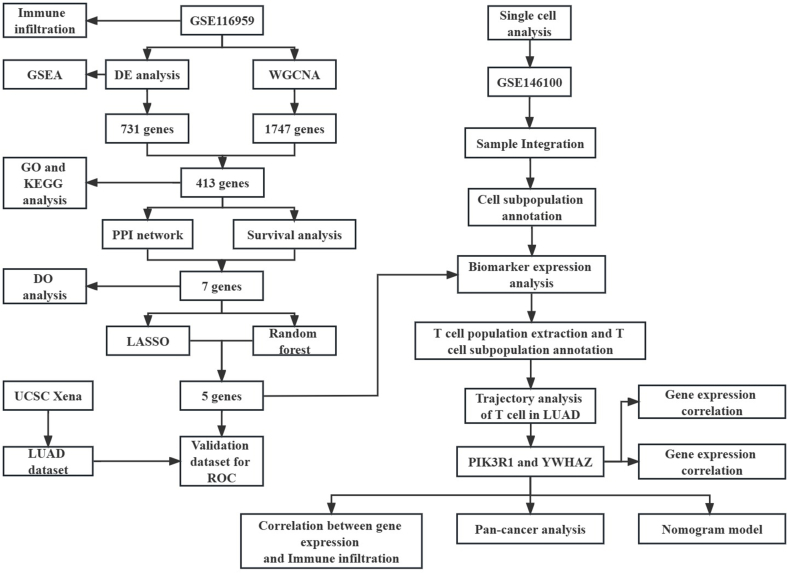
The flow chart for the multiomics method.

**Figure S2:**
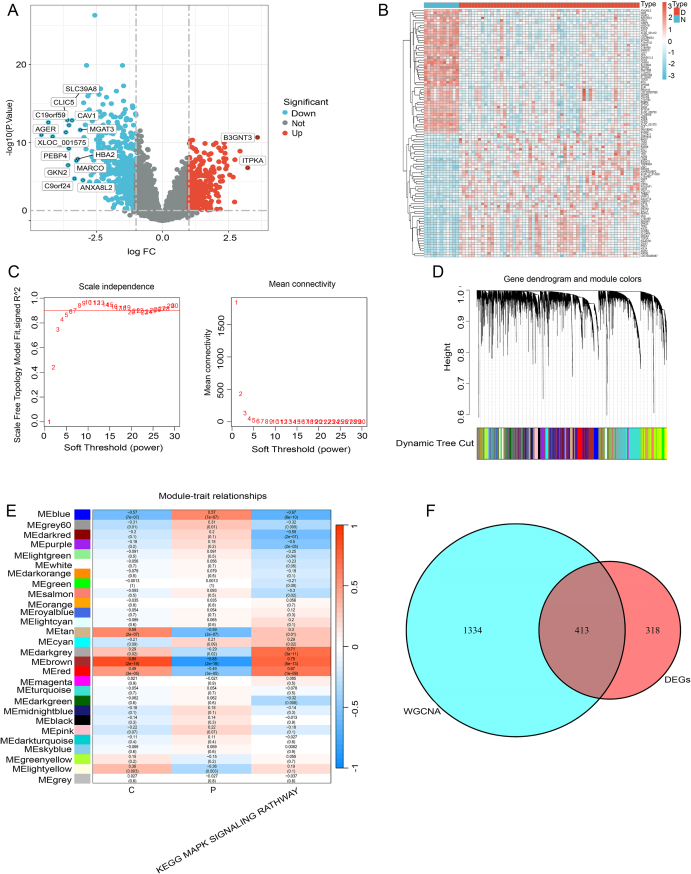
Identification of DEGs and MAPK pathway-related hub module genes. **(A)** The volcano plot showing the expression characteristics of DEGs, where red represents gene up-regulation in the LUAD group, and blue represents gene up-regulation in the control group. **(B)** The heat map showing the expression of the top 50 DEGs in the sample. **(C)** Scale-free fit index (proportional independence, y-axis) as a function of soft threshold power (x-axis) (left) and mean connectivity (degrees, y-axis) as a function of soft threshold power (x-axis) (right). **(D)** LUAD module clustering dendrogram based on a dissimilarity measure (1-TOM). The colored rows below the dendrogram indicate the module assignment identified by dynamic tree shearing. **(E)** Module–feature correlations. Each column corresponds to a clinical feature, whereas individual rows in the heat map correspond to an ME. Individual cells contain the corresponding *P*-value and correlation coefficient. **(F)** WGCNA module genes mapped to DEGs. DEGs, differentially expressed genes; LUAD, lung adenocarcinoma.

**Figure S3:**
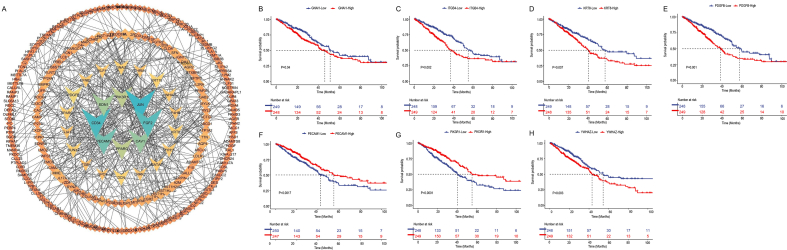
Screening for survival-related genes. **(A)** The protein-protein interaction network. “V-shaped” patterns represent genes with degree ≥10. **(B–H)** The Kaplan-Meier curves for seven genes associated with survival in lung adenocarcinoma (LUAD) patients, namely, GNAI1, ITGB4, KRT8, PDGFB, PECAM1, PIK3R1, and YWHAZ.

**Figure S4:**
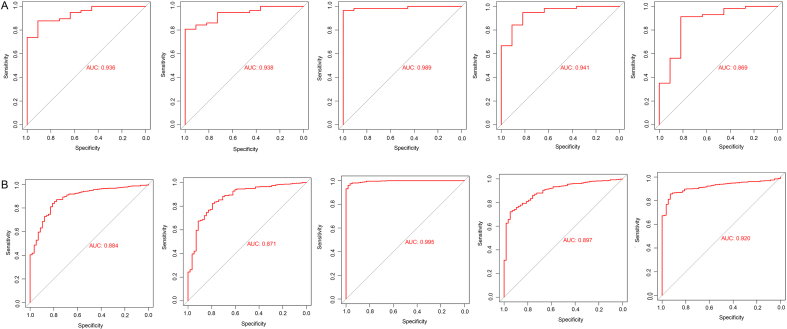
ROC validation of potential biomarkers. **(A)** The ROC curve showing potential biomarkers in the GSE116959 dataset. **(B)** The ROC curve showing potential biomarkers in the TCGA dataset. ROC, receiver operating characteristic.

**Figure S5:**
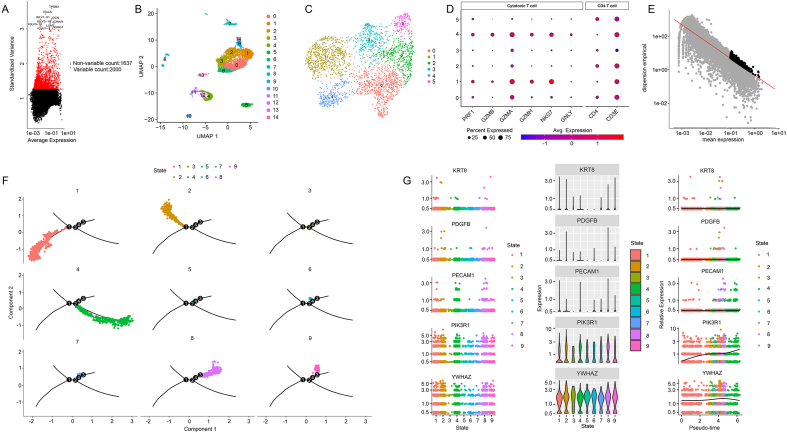
Single-cell analysis results. **(A)** The variance plot showed 16,379 genes in all cells. The red dots represent the top 2000 highly variable genes. **(B)** 15 clusters were visualized based on the UMAP algorithm. **(C)** Six clusters of T cells were visualized based on the UMAP algorithm. **(D)** Expression of marker genes in different clusters of T cells. **(E)** Dimensionality reduction in trajectory analysis. **(F)** The Monocle 2 trajectory plot showing the states of T cells. Four crucial proposed time points divided T cells into nine states. **(G)** Variation in expression of potential biomarkers in different T cell states.

**Figure S6:**
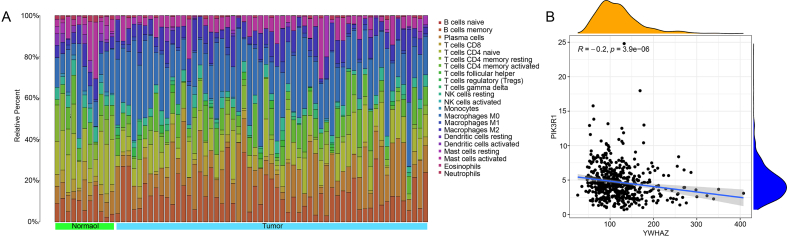
Immune cell infiltration analysis and gene expression correlation analysis. **(A)** The bar plot showing the proportion of 22 types of immune cells. **(B)** Correlation of PIK3R1 and YWHAZ expression in lung adenocarcinoma (LUAD).
