# Supplementary material for: Exploring biomarkers of MAPK pathway co-expression in lung adenocarcinoma and their functions based on machine learning algorithms and single-cell analysis
Source: Genes Dis. 2024 Jan 26;12(1):101222. doi: 10.1016/j.gendis.2024.101222 (PMC11472232; doi:10.1016/j.gendis.2024.101222)
Supplement: Multimedia component 1 [file mmc1.docx]

**Materials and methods**

**Acquisition of transcriptome data and** **scRNA-seq data**

Gene expression data were obtained from the NCBI (National Center for Biotechnology Information) Gene Expression Omnibus public database (GEO) (http://www.ncbi.nlm.nih.gov/geo/). The GEO dataset, GSE116959, was chosen among the datasets based on the following criteria: 1. only includes datasets of Homo sapiens, 2.  include datasets produced within 15 years, exclude datasets without detailed descriptions. GSE116959 contained RNA expression data annotated by GPL17077 in the tumor tissues, which included 57 LUAD samples and 11 ND controls. The scRNA-seq data of 2 LUAD samples (W1 and W2) of GSE146100 were downloaded from the GEO database.

**Identification of differentially expressed genes (DEGs)**

Software R (version 4.2.2, <https://www.r-project.org/>) and “limma” package were used to perform the significance analysis of DEGs between LUAD samples and normal samples. Firstly, we used the normalizeBetweenArrays function in the “limma” package to normalize the samples. Then, Genes with *P_adj* < 0.05 and *|Log2(fold change)|* > 1 were considered as DEGs.

**Screening of co-expressed genes of MAPK pathways by weighted gene co-expression network analysis**

Using the GSEA database, we selected MAPK signaling pathway (https://www.gsea-msigdb.org/gsea/msigdb/human/geneset/KEGG_MAPK_SIGNALING_PATHWAY) that are associated with many types of cancer. To explore the co-expression relationships between genes and between genes and the MAPK signaling pathways, a gene co-expression network was constructed using the "WGCNA" package in R software ^16^. The similarity matrix was constructed by calculating the correlation coefficient between each gene pair. We chose a proper soft threshold to transform the similarity matrix into an adjacency matrix, which ensures the construction of a scale-free network. Thereafter, a topological overlap matrix (TOM) was created to measure the average network connectivity of each gene. A module is a set of genes whose expression is highly correlated, and these highly correlated genes can be identified and grouped into a module using the hierarchical clustering and Dynamic Tree Cut methods. Based on the “blockwiseModules” function, we use the dynamic tree cutting method to group genes with similar expression profiles into the same modules. The different modules are depicted in different colors. Module eigengene (ME) is the first principal component of each module and represent the gene expression profile of each module. In addition, we calculated module membership (MM), gene significance (GS), and modules associated with clinical features, and then visualized the network of feature genes. We selected WGCNA-derived modular genes that were significantly associated with MAPK signaling pathways in LUAD. We used the intersection between DEGs and WGCNA-derived significant module genes from GSE116959 to obtain potential gene targets of LUAD.

**GO term and KEGG pathway enrichment analysis and PPI network generation**

The “clusterProfiler” R package was used to perform Gene Ontology (GO) and Kyoto Encyclopedia of Genes and Genomes (KEGG) analyses of the functions of the genes obtained from the intersection. *P* < 0.05 was selected as the threshold for significant enrichment. Additionally, the PPI network was created and analyzed with the STRING database to elucidate the correlation between LUAD and the MAPK pathway and to obtain insights into the interactions between target genes of LUAD and genes related to the MAPK pathway. The results of the interactions were visualized with Cytoscape ^17^.

**Survival analysis of target genes and Disease Ontology (DO) analysis**

The 26 genes with *Degree values* > 10 were selected as target genes. Subsequently, we performed survival analysis on the target genes. We download gene expression data and survival data from the UCSC database (<https://xenabrowser.net)> for LUAD samples. Then, survival analysis was performed using the “survival” package and we defined gene expression greater than the median as high expression and gene expression less than the median as low expression. *P* < 0.05 was considered statistically significant. DO enrichment analysis was used to analyze the association of genes with diseases and DO analysis was performed for genes significantly associated with survival time in LUAD.

**Potential biomarkers screening for LUAD patients by machine learning algorithms**

We used two machine learning algorithms, including LASSO regression analysis and random forest analysis. First, we used the "glmnet" package to perform the LASSO regression analysis, and the genes obtained by the algorithm were subjected to a subsequent random forest analysis. The "randomForest" package was used to perform the random forest analysis and the genes with "MeanDecreaseGini>2" were selected for the subsequent analysis. The crossover genes obtained using both machine learning algorithms were considered as potential biomarkers for LUAD patients. Finally, based on the "pROC" package, we used the receiver operating characteristic curve (ROC) and the area under the ROC curve to assess the diagnostic efficacy of the model.

**Data integration and the dimensionality reduction**

The Seurat object with gene expression data from individual samples was processed with the Read.delim() and CreateSeuratObject() functions. Low quality cells were removed. For each sample, the data is normalized using the NormalizeData() function, with the “normalization.method” parameter set to "LogNormalize" and the “scale.factor” set to 10000. Prior to performing principal component analysis (PCA), we identified the top 2000 highly variable genes (HVG) from the normalized expression matrix. Then, we use the harmony package to remove the batch effects.

**Cell-clustering and annotation**

Clustering analysis was done based on the "FindClusters" and "FindNeighbors" functions in the “Seurat” package. The identified clusters were visualized on the 2D map produced with the UMAP method. Subsequently, we used the HumanPrimaryCellAtlasData() function to obtain a reference dataset from the Human Primary Cell Atlas, based on which cell subclusters were annotated using “singleR” package.

**T cell subclusters annotation and trajectory analysis of single-cells**Single-cell pseudotime trajectory of T cell was generated using the “Monocle” package (v2.22.0) in R software (version 4.1.3). Initially, T cells from the annotated cell subclusters were selected for T cell subclusters annotation and proposed time analysis from the W1 type samples, which were a tumor sample that did not respond to the pembrolizumab. The T cell groups were annotated based on the well-known cellular markers from the literature ^18^. Detailed information of the markers was provided in Table S1. Then, we applied the “newCellDataSet” function to create an object. Genes with the mean expression ≥ 0.1 were used in the trajectory analysis. The reduceDimension() function was applied to reduce the dimension with parameters reduction_method = "DDRTree" and max_components = 2. Followed by this, we used the plot_cell_trajectory() function to cells for sorting and visualization. We also used the functions plot_genes_jitter(), plot_genes_violin(), and plot_genes_in_pseudotimes() to calculate the expression of the five potential biomarkers changed along with the states. We calculated which of the potential biomarkers that changed along with the pseudotime by using the differentialGeneTest() function with parameters set to *qval* < 0.1 and visualized them with “plot_pseudotime_heatmap” function.

**Analysis of the correlation between immune cell infiltration and *PIK3R1* and *YWHAZ* expression**

Since *PIK3R1* and *YWHAZ* are expressed in a high percentage of T cells and both may have a non-negligible effect on LUAD, we selected *PIK3R1* and *YWHAZ* for subsequent pan-cancer analysis and network and nomogram construction. TIMER is a platform that enables systematic analysis of the level of infiltrating immune cells in various cancers ^19^. Based on TIMER, we analyzed the correlation between two of the marker genes (*PIK3R1* and *YWHAZ*) that are highly expressed in immune cells and the level of infiltration of various immune cells in LUAD.

**Immune infiltration and immune cell correlation analyses**

The "CIBERSORTx" website (a website developed based on CIBERSORT) was used to analyze the expression status of 22 different immune cells in LUAD patients' tumor tissue. Further, the differences in the proportion of various immune cell infiltrations in LUAD tissues and normal lung tissues were compared. Then, the 22 immune cells infiltrating into the lung tissue were correlated to further understand the changes occurring in the immune system in the lung tissue of LUAD patients.

**Pan-cancer analysis and correlation analysis of *PIK3R1* and *YWHAZ* expression**

We downloaded gene expression data from the UCSC database for various cancers. Then we analyzed the expression of *PIK3R1* gene in various cancers and control samples. Finally, we used R software to visualize the analysis results.

**TF-mRNA network construction**

TF-mRNA networks help us understand the interconnection of gene transcription processes with transcription factors, as well as to explore the mechanisms of expression correlation between individual genes. The NetworkAnalyst platform (https://www.networkanalyst.ca) can be used to analyze the interactions of genes, transcription factors (TFs), drugs and other factors. Based on the NetworkAnalyst platform, we constructed the *PIK3R1* and *YWHAZ* and TFs interaction network.

**Pan-cancer analysis of *PIK3R1* and *YWHAZ* and the level of immune cell infiltration**

To understand the effect of *PIK3R1* and *YWHAZ* on immune cells in various cancers, we analyzed *PIK3R1* and *YWHAZ* in the TIMER database in relation to the level of immune cell infiltration in a variety of common cancers. We collated a large amount of data and visualized the collated data using the "ggplot2" package.

**Construction of the potential biomarkers-based nomogram**

Nomogram was used to predict the relationship between gene expression and prevalence in a population. We evaluated whether *PIK3R1* and *YWHAZ* are potentially positive for the diagnosis of LUAD in the clinical setting by nomogram. The "rms" package was used to construct a nomogram based on the *PIK3R1* and *YWHAZ*. Subsequently, we predicted the prevalence of LUAD in the population based on the expression of the two potential biomarkers.
