# Supplementary material for: Exploring biomarkers of MAPK pathway co-expression in lung adenocarcinoma and their functions based on machine learning algorithms and single-cell analysis
Source: Genes Dis. 2024 Jan 26;12(1):101222. doi: 10.1016/j.gendis.2024.101222 (PMC11472232; doi:10.1016/j.gendis.2024.101222)
Supplement: Multimedia component 2 [file mmc2.docx]

**Title:** Exploring biomarkers of MAPK pathway co-expression in lung adenocarcinoma and their functions based on machine learning algorithms and single-cell analysis.

**Authors and Affiliations:** Mingkai Lin^a^, Ruoyi Zheng^a^, Peixian Liang^b^, Jiayang Huang^a^, Xintong Ke^c^, Wenjing Zhang^d,**^, Pei Shang^a,*^

^a^ *Breast Center, Department of General Surgery, Nanfang Hospital, Southern Medical University, Guangzhou 510515, China*

*^b^ Department of Ultrasound, the Second Affiliated Hospital of Guangzhou Medical University, Guangzhou 510515, China*

^c^ *College of Teacher’s Education, Guangdong University of Education, Guangzhou 510515, China*

^d^ *Department of Respiratory Medicine, Puyang People's Hospital, Henan, 457000, China*

^*^ Corresponding authors.

Breast Center, Department of General Surgery, Nanfang Hospital, Southern Medical University, Guangzhou 510515, China.

^**^ Corresponding authors.

Department of Respiratory Medicine, Puyang People's Hospital, Henan, 457000, China

*E-mail address:* [andy.shang666@hotmail.com](mailto:andy.shang666@hotmail.com) (P. Shang), [15649736886@163.com](mailto:15649736886@163.com) (W. Zhang).

**Introduction:** Lung adenocarcinoma (LUAD) is the most common histological subtype of primary lung cancer nowadays, and is characterized by a variety of gene mutations and altered gene expression profiles, and therefore studies at the gene level of LUAD can help to elucidate the mechanisms of the occurrence and progression of LUAD and the direction of treatment. The MAPK pathway, a key pathway that influences the development of many tumors, also mediates the progression of LUAD. Studies suggest that STAMBP, CMaf-inducing protein, and KIAA1429 promotes LUAD proliferation by regulating EGFR/MAPK, MAPK/ERK, and JNK/MAPK pathway respectively. A better understanding of MAPK pathways is critical to determine whether these pathways are promising therapeutic targets for LUAD. In addition, the immune microenvironment of LUAD tissues is also involved in controlling the proliferation and metastasis of tumor cells.

**Objective and Research Question:** Our study aims to unite the functions of LUAD-related pathways and immune microenvironment based on massive bioinformatic data to excavate the potential gene networks affecting LUAD occurrence, progression, prognosis and treatment. The workflow of our study is shown in Figure S1.

**Methods:** First, we downloaded various types of data from GEO and TCGA databases, including gene expression data, single cell data and survival data. We used the "limma" package in R software to screen differential genes and the "WGCNA" package to screen genes highly related to the MAPK pathway, in order to screen more specific LUAD-related genes from a large number of genes. Subsequently, the screened genes were subjected to functional enrichment analysis, protein-protein network construction and Kaplan-Meier analysis to screen the genes with more research value and prognostic relevance. The above three analyses were performed using the "clusterProfiler" R package, the STRING database and the "survival" R package, respectively. Finally, the prognosis-related genes were further downscaled by LASSO and Random Forest algorithms. Through the above multiple analysis methods, we screened tens of thousands of genes and identified five genes, which constitute a gene network specific to LUAD. These five genes are KRT8, PDGFB, PECAM1, PIK3R1 and YWHAZ. In order to explore the expression characteristics and biological functions of the above five genes in various types of cells in tumor tissues, we performed single-cell data analysis. First, we preprocessed the single-cell data using a variety of functions from the “Seurat package”. After annotating the cell subclusters of various cells using the "singleR" package, we screened the T cells for subsequent single-cell trajectory analysis. The trajectory analysis was performed by the “Monocle” package, and then the "plot_pseudotime_heatmap" function of this package was used to explore the expression changes of the five biomarkers screened above with the development of T cells. The TIMER platform, as a database containing a large amount of gene information, was used as a basis for us to perform pan-cancer analysis of individual genes in order to further investigate the pan-cancer efficacy of genes. As a final step, we constructed column-line plots to illustrate the clinical significance of combining different genes for LUAD and further validated these results by plotting calibration curves, decision curves and clinical impact curves.

**Results:** 731 differential genes associated with LUAD were screened by differential gene analysis. MAPK pathway co-expressed genes were screened by WGCNA analysis. The intersection of the above two gene sets was taken to obtain the final 413 subgene set. The results of pathway enrichment analysis showed that these genes were associated with tissue migration and lesion adhesion, among others. Subsequently, through PPI network construction, survival analysis and two machine learning analyses, we screened the five potential biomarkers in the subgene set that were most valuable for LUAD, namely KRT8, PDGFB, PECAM1, PIK3R1 and YWHAZ. in order to explore the predictive value of each biomarker for LUAD, we performed the plotting of ROC curves. roc curves showed that the AUC values for each of the above biomarkers were 0.936, 0.938, 0.989, 0.941, and 0.869 in the test set and 0.884, 0.871, 0.995, 0.897, and 0.920 in the validation set in the TCGA database, respectively. In order to illustrate the status of the five biomarkers in various types of cells of tumor tissues, we identified seven cell types from the single-cell dataset GSE146100, namely B cells, epithelial cells, macrophages, monocytes, NK cells, smooth muscle cells and T cells. Then, we analyzed the expression of potential biomarkers KRT8, PDGFB, PECAM1, PIK3R1 and YWHAZ in 7 types of cells of LUAD samples. The results showed that PECAM1 was more highly expressed in monocytes, macrophages and smooth muscle cells, PIK3R1 in T cells, KRT8 in epithelial cells and YWHAZ in lymphocytes. Since T cells, as key immune cells in the tumor microenvironment species, influence the proliferation and metastasis of tumor cells, we extracted T cells and subsequently analyzed the differences in the expression of the above biomarkers in different species of T cells, with PIK3R1 being more highly expressed in CTLs and YWHAZ being more highly expressed in CD3-CD4- T cells on average. Through trajectory analysis, we can understand the unique biological information of different stages of cell growth or differentiation. The trajectory analysis illustrated that as T cells differentiated and matured, significant expression changes appeared in YWHAZ, PDGFB, PIK3R1 and PECAM1. PIK3R1 and PECAM1 showed a gradual increase in expression with the differentiation and maturation of T cells, and the expression peaks of YWHAZ and PDGFB appeared in the process of T cell differentiation and maturation. To further investigate the influence of potential immune-related genes on the tumor immune microenvironment, we selected PIK3R1 and YWHAZ, which have more specific high expression in immune cells. Pan-cancer analysis of PIK3R1 and YWHAZ immune-related showed that PIK3R1 was highly expressed in B cells. To infer the accuracy of the combined PIK3R1 and YWHAZ model for predicting LUAD in order to further elaborate the specificity of PIK3R1 and YWHAZ for LUAD, we constructed column-line plots. The column plot showed that the median accuracy of the combined PIK3R1 and YWHAZ model for the prediction of LUAD was 98.5%.

**Research significance:** In order to explore the biological characteristics of key genes in LUAD, this study focused on screening biomarkers for LUAD. The results showed that KRT8, PDGFB, PECAM1, PIK3R1 and YWHAZ were potential biomarkers of LUAD, and these genes were closely associated with the prognosis of LUAD patients. Our study reveals that PIK3R1 and YWHAZ are closely associated with a variety of immune cells in the tumor immune microenvironment, and their expression is significantly altered during T cell differentiation and maturation. In addition, we pointed out that PIK3R1 could predict the differentiation of T cells to cytotoxic T cells, which provided a new reference direction for the study of the development of drugs targeting T cells to inhibit tumor proliferation in LUAD. Moreover, we initially explored the accuracy of the combined model of PIK3R1 and YWHAZ for predicting LUAD by constructing a column-line diagram, and the results confirmed the high accuracy of the model. And last, single-gene pan-cancer analysis showed that the effects of PIK3R1 and YWHAZ on immune cells of other tumors, such as thyroid cancer, should not be neglected.


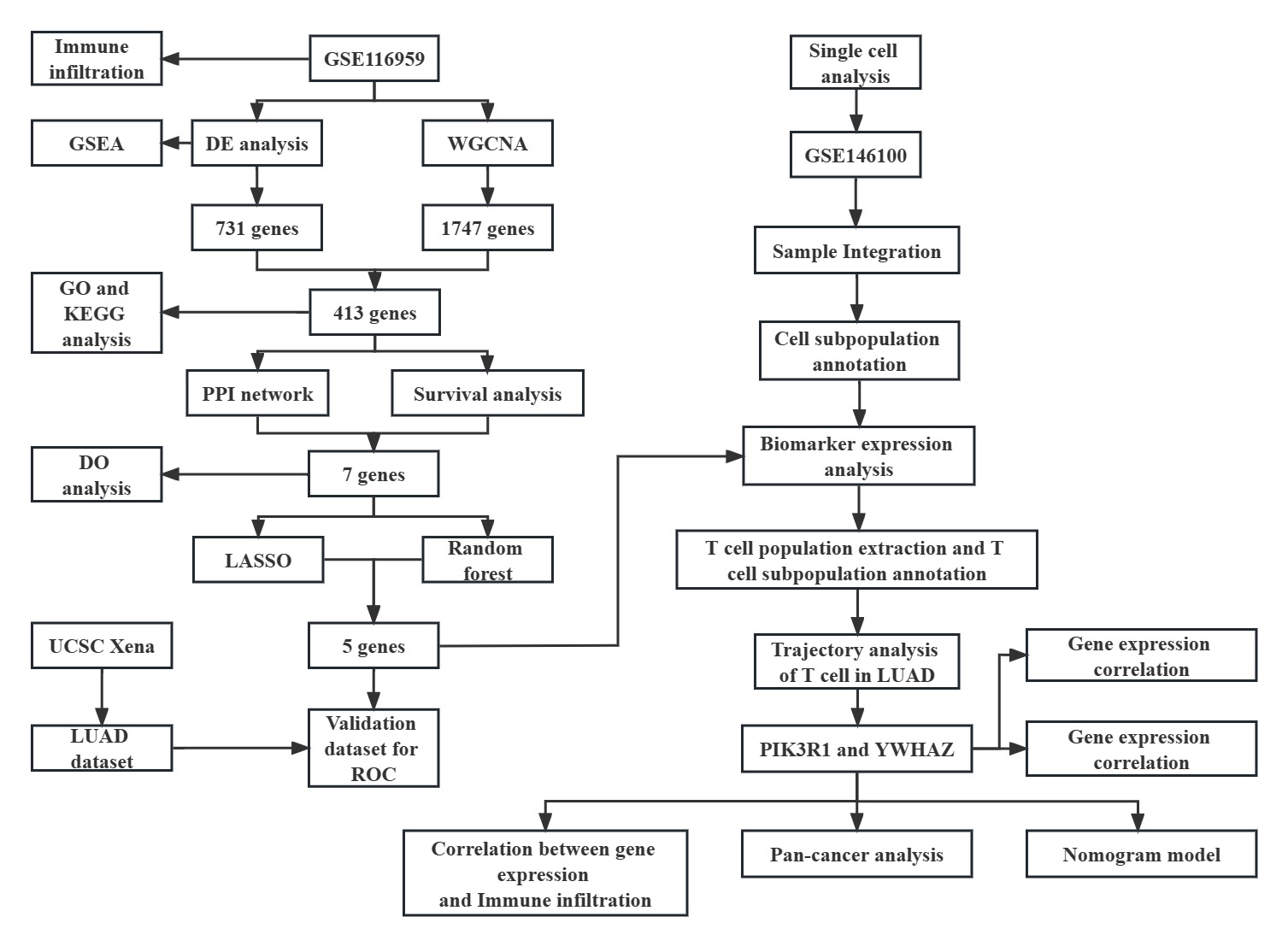


**Figure S1** The flow chart for multiomics method.
