# Supplementary material for: Exploring biomarkers of MAPK pathway co-expression in lung adenocarcinoma and their functions based on machine learning algorithms and single-cell analysis
Source: Genes Dis. 2024 Jan 26;12(1):101222. doi: 10.1016/j.gendis.2024.101222 (PMC11472232; doi:10.1016/j.gendis.2024.101222)
Supplement: Multimedia component 3 [file mmc3.docx]

**Table S1** T-cell subclusters and their marker genes.

| Cell type | Marker genes |
| --- | --- |
| CTL (cytotoxic T cell) | PRF1, GZMB, GZMA, GZMH, NKG7, GNLY |
| CD4 T cell | CD4, CD3E |
